# Development and Validation of Intracranial Thrombus Segmentation on CT Angiography in Patients with Acute Ischemic Stroke

**DOI:** 10.1371/journal.pone.0101985

**Published:** 2014-07-17

**Authors:** Emilie M. M. Santos, Henk A. Marquering, Olvert A. Berkhemer, Wim H. van Zwam, Aad van der Lugt, Charles B. Majoie, Wiro J. Niessen, on behalf of the MR CLEAN investigators

**Affiliations:** 1 Dept. of Radiology, Erasmus MC, Rotterdam, the Netherlands; 2 Dept. of Medical Informatics, Erasmus MC, Rotterdam, the Netherlands; 3 Dept. of Radiology, AMC, Amsterdam, the Netherlands; 4 Dept. of Biomedical Engineering and Physics, AMC, Amsterdam, the Netherlands; 5 Dept. of Radiology, Maastricht University Medical Centre, Maastricht, the Netherlands; 6 Faculty of Applied Sciences, Delft University of Technology, Delft, the Netherlands; INSERM U894, Centre de Psychiatrie et Neurosciences, Hopital Sainte-Anne and Université Paris 5, France

## Abstract

**Background and Purpose:**

Thrombus characterization is increasingly considered important in predicting treatment success for patients with acute ischemic stroke. The lack of intensity contrast between thrombus and surrounding tissue in CT images makes manual delineation a difficult and time consuming task. Our aim was to develop an automated method for thrombus measurement on CT angiography and validate it against manual delineation.

**Materials and Methods:**

Automated thrombus segmentation was achieved using image intensity and a vascular shape prior derived from the segmentation of the contralateral artery. In 53 patients with acute ischemic stroke due to proximal intracranial arterial occlusion, automated length and volume measurements were performed. Accuracy was assessed by comparison with inter-observer variation of manual delineations using intraclass correlation coefficients and Bland–Altman analyses.

**Results:**

The automated method successfully segmented the thrombus for all 53 patients. The intraclass correlation of automated and manual length and volume measurements were 0.89 and 0.84. Bland-Altman analyses yielded a bias (limits of agreement) of −0.4 (−8.8, 7.7) mm and 8 (−126, 141) mm^3^ for length and volume, respectively. This was comparable to the best interobserver agreement, with an intraclass correlation coefficients of 0.90 and 0.85 and a bias (limits of agreement) of −0.1 (−11.2, 10.9) mm and −17 (−216, 185) mm^3^.

**Conclusions:**

The method facilitates automated thrombus segmentation for accurate length and volume measurements, is relatively fast and requires minimal user input, while being insensitive to high hematocrit levels and vascular calcifications. Furthermore, it has the potential to assess thrombus characteristics of low-density thrombi.

## Introduction

Treatment of acute ischemic stroke aims to reopen the cerebral arteries and restore cerebral perfusion. Numerous studies have related image characteristics of thrombus (size, location, and density) measured on CT, with treatment success [1]–[5]. Although in various studies, positive associations have been reported, their predictive value for treatment success is still under debate. The methods that were used to assess thrombus characteristics vary widely. Therefore, thrombus characteristics and outcome reported in different studies cannot be compared. An automated, objective, and reliable measurement tool would be of great value in investigating the association between thrombus characteristics and treatment outcome, and in facilitating an objective comparison tool between studies.

The composition of thrombus may vary. White clots consist of platelets and fibrin, whereas red clots are erythrocyte-rich [6]. Clots dominantly composed of red blood cells can be detected on non-contrast CT (NCCT), where it is expressed as a hyperdense artery sign. Using an automated method, Riedel & al [7] measured hyperdense middle cerebral artery thrombus length on NCCT with mean deviations from the reference values and limits of agreement of 0.1 mm±0.7 mm using <2.5 mm slice images, demonstrating that hyperdense thrombi can be measured accurately. The reported prevalence of hyperdense thrombi in patients with ischemic stroke ranges from 2% to 58% depending on thrombus location [8]–[10]. A challenge for hyperdense thrombus segmentation techniques on NCCT data is the possible presence of hyperdensities in intracranial vessels due to calcified arterial walls or high hematocrit [11] that mimics hyperdense thrombus. Because of the lack of contrast between low-density thrombi and surrounding tissue on NCCT, low-density thrombi can only be visualized and quantified in CTA images using the absence of contrast-enhanced lumen. However, this approach requires sufficient retrograde filling of the occluded vessel via collateral arteries, which occurs in 51% to 87% of the cases [5], [12] and is related to the scan protocol [13]. The Clot Burden Score has been proposed as an alternative approach for the assessment of thrombus extent. It is a semi-quantitative measurement that assesses the volume and location of the thrombus on CTA [1]. However, the Clot Burden Score is a rather crude measure since it does not include the actual length and volume of the thrombus, which may be predictors of treatment outcome.

Our aim was to develop and validate an automated method to detect and quantify thrombus on CTA, requiring minimal user input. The method exploits the absence of contrast at the site of thrombus by using anatomical information from the contralateral arteries. The method was validated for high density thrombus by comparison with manual delineation of thrombus visible on NCCT.

## Materials and Methods

### Patients

We retrospectively collected baseline thin slice (<2.5 mm) NCCT and CTA image data from the Dutch multi-center image database of the MR CLEAN clinical trial (Registries: NTR1804/ISRCTN10888758; protocol available at www.mrclean-trial.org). CTA was used for the automated thrombus segmentation method. Thin slice NCCT was used in this study for the manual delineation of thrombus to validate the automated method. Therefore both baseline thin slice NCCT and CTA was required for a patient to be included. All patients had a clinical diagnosis of acute ischemic stroke due to proximal intracranial arterial occlusion with a deficit on NIHSS of 2 points or more; occlusion of the distal internal, middle, or anterior cerebral arteries, the possibility to start treatment within 6 hours from onset, and age 18 or over. Exclusion criteria for this study were low quality scans, and incomplete volume of interest.

### Ethic statement

This retrospective study has been approved by the Medical and Ethical Review Committee (Medisch Ethische Toetsings Commissie; http://www.erasmusmc.nl/commissies/metc/) from Erasmus MC, Rotterdam, The Netherlands. Moreover, all participating centers providing image data received approval from their local medical ethical committee. All patient records, informations and images were anonymized and de-identified prior to analysis. All patients or legal representatives signed informed consent.

### Automated Thrombus Segmentation Method

**Figure 1:**
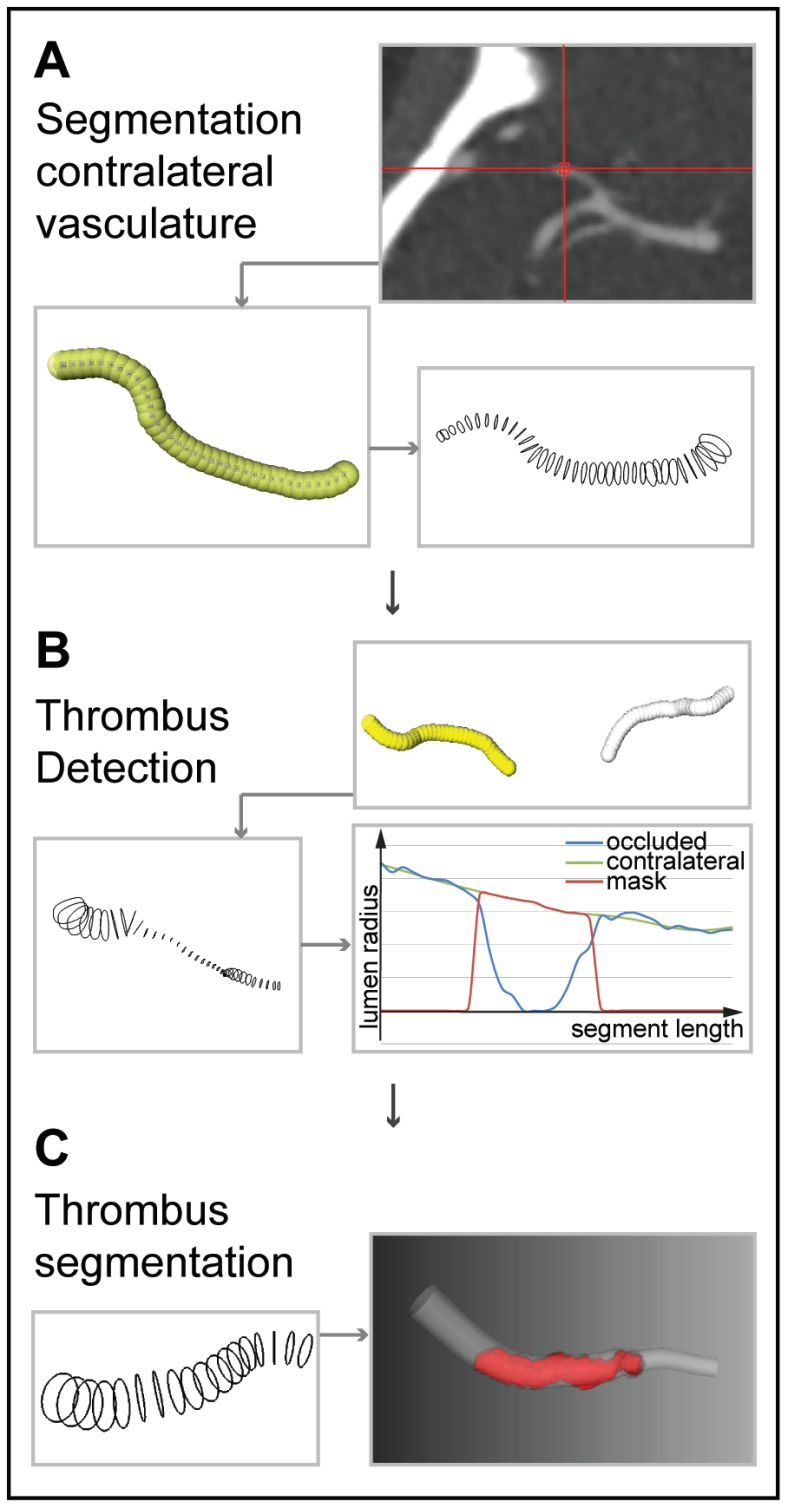
Illustration of the pipeline of the automated method. (A) The segmentation of the contralateral vasculature is started after manual seeds point placement. The lumen is segmented using a graph cut segmentation.(B) The thrombus is detected by mirroring the centerline and a Bspline registration followed by the segmentation of the occluded segment. Distal and proximal thrombus position is detected using the lumen radii.(C) Thrombus is segmented using tubular masking and region growing.

The proposed automated thrombus segmentation consists of three steps: (1) segmentation of the contralateral vasculature; (2) creation of a mask in the occluded arterial segment by mapping the contralateral segmentation using mirror symmetry; (3) segmentation of the thrombus using intensity based region growing. The image processing pipeline was implemented in MeVisLab [14] (www.mevislab.de), the different steps are visualized in Figure 1 and the main parameters used are presented in the Table 1.

**Table 1:** Image segmentation parameters and their corresponding values.

Parameters	Values (unit)
Vesselness filter (σ_low_, σ_up_)	1.0, 3.5
Graph cut (σi_nside_, σ_outside_)	20, 20 (Hounsfield unit)
Radial Reg. (fraction_outside_, P_outside_, blurσ_distance_)	0.75, 0.5, 2 (mm)
Weighed Gaussian Reg. (Iteration, σ_radius_)	2, 0.5 (mm)
3D-BSpline registration (Grid spacing, grey level)	2 (mm), 32 (bit)
Mathematical morphology (opening, dilatation (3D kernel (2n+1)))	n = 1(voxel), n = 1(voxel)

The contralateral vasculature was segmented in two steps. First, proximal and distal seed points were placed manually ensuring that they covered the contralateral thrombus. Next, a curve was created through the contralateral vasculature using an optimal path calculation on a cost image generated with a vesselness filter [15], [16]. Using this curve as an initial estimation of the centerline, cross-sectional lumen boundary contours were obtained using a graph-cut technique with kernel regression [17]. A refined centerline was generated by connecting all centers of mass of the lumen boundary contours. Subsequently, the vessel radius along the centerline was defined as the average distance from each center of mass to their respective lumen boundary contour. To correct for large variations in radii along the center line, which may be the result of blooming artifacts caused by calcifications, the radius along the centerline was smoothed with a weighted Gaussian kernel [18].

An initial estimate of the centerline of the occluded arterial segment was obtained by mirroring the contralateral centerline. This estimation was improved by performing a 3D-Bspline registration using Elastix [19] (www.elastix.isi.uu.nl), of the mirrored region of interest of the contralateral side with the region of interest of the occluded arterial segment. The mirrored centerline was used as an initialization for graph-cut segmentation with kernel regression. As it was done for the contralateral vasculature, the lumen radii along the centerline were estimated. Because the intensity of thrombi (<80 Hounsfield unit) was much lower than contrast enhanced lumen (∼150 to 300 Hounsfield unit), this segmentation never includes the thrombus. As a result, the estimated lumen radius at the position of the thrombus was much smaller than the radius at the contralateral artery. The start and end positions where the estimated radius was smaller than 0.05 times the radius of the contralateral site were used as an initial estimation of the proximal and distal location of the occlusion. In some cases, due to expected insufficient contrast flow from collaterals, the method used the end part of the vasculature as distal reference for the masking step.

In the next step the thrombus was segmented. First, a mask was generated to exclude the surrounding tissues and the contrast enhanced lumen that should not be considered in the intensity based region growing segmentation. The mask was defined as a tube around the centerline of the occluded arterial segment with the radius of the contralateral arterial segment. The mask ranges from the estimated proximal and distal location of the occlusion. Intensity based region growing for thrombus segmentation was initialized at the centerline approximately 0.5 mm distal to the estimated proximal location of the occlusion. The region growing method requires two thresholds. The maximum threshold was defined as the average intensity along the contralateral centerline subtracted with the standard deviation of the intensity values along the same centerline. The minimum threshold was defined as the minimal intensity along the occluded arterial centerline in the estimated thrombus added with the standard deviation of the intensity values. A mathematical morphological opening was performed to smooth the contours of the thrombus. Length and volume are automatically measured from the resulting segmentation. The volume was determined by computing the number of segmented voxels multiplied by the size of a voxel and the length was measured from the extremities of the segmented volume along the centerline.

### Manual delineations

**Figure 2:**
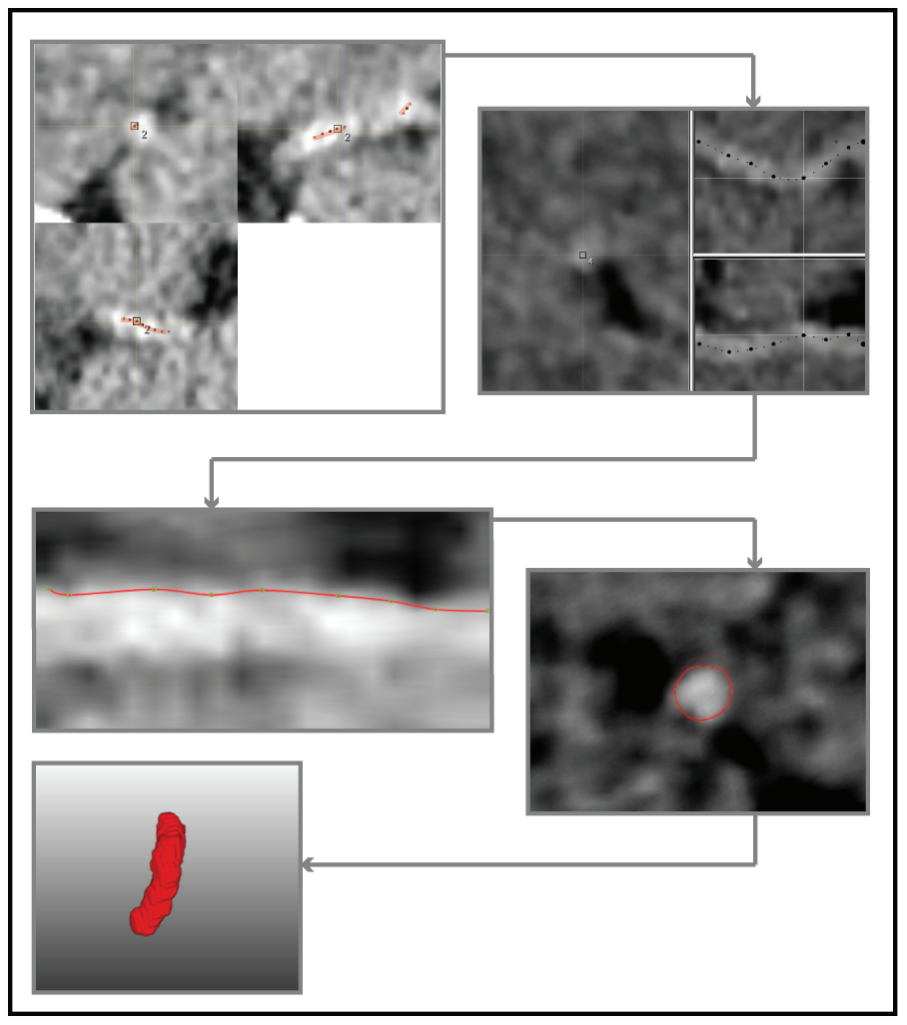
Illustration of the manual segmentation process. A centerline is drawn in orthogonal views and subsequently corrected using MPR viewing. The contours of the thrombus are drawn first in a longitudinal view and subsequently corrected using on a transversal view.

Manual delineations of the thrombi were used to assess the accuracy of the automated method. Three neuroradiologists (CBM, WHvZ and AvdL), each with more than 10 years of experience, performed the manual delineations. The observers only had access to baseline NCCT and CTA images of the patient during thrombus delineation and were blinded from all clinical information apart from the symptom side. To assist manual delineation, a custom made tool was developed in MeVisLab. With this tool, a manual centerline was first obtained by interpolating a polynomial curve through manually placed points in the thrombus on the baseline NCCT scan. Next, the thrombus contours were delineated on a stretched-vessel view through the occluded arterial segment. Thrombus volume and length were subsequently determined from these contours. The different steps of the manual delineation pipeline are visualized in Figure 2. Because low-density thrombi were not visible on the NCCT image data, only high-density thrombi were selected for validation. The time needed for automated segmentation and manual delineation was recorded.

### Statistical analysis

To assess the accuracy, automated length and volume measurements were compared with the manual delineations performed by observer 1. Because of the lack of a reference standard, we compared variation between the automated method and observer 1 with the interobserver variation of the manual delineation. To assess the interobserver agreement, 20 datasets were delineated by both observer 1 and 2, and 20 other datasets were delineated by both observer 1 and 3. SPSS (SPSS version 20.0) was used to calculate the intraclass correlation coefficient (ICC) and to perform a Bland & Altman analyses.

## Results

**Figure 3:**
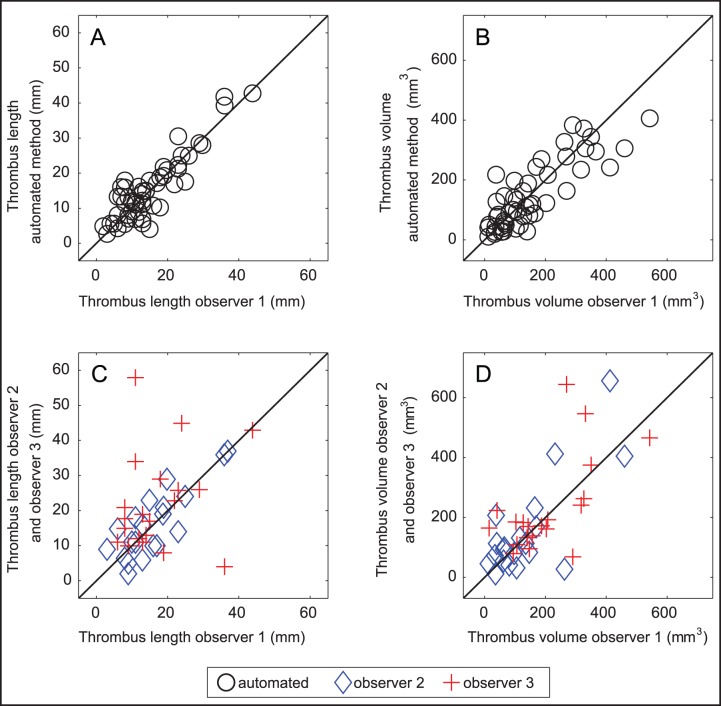
Scatter plot of thrombus length and volume of observer 1 and automated method (A, B). Scatter plot of thrombus length and volume of observer1 and observer 2 and 3 (C, D). The solid line represents the identity line.

**Figure 4:**
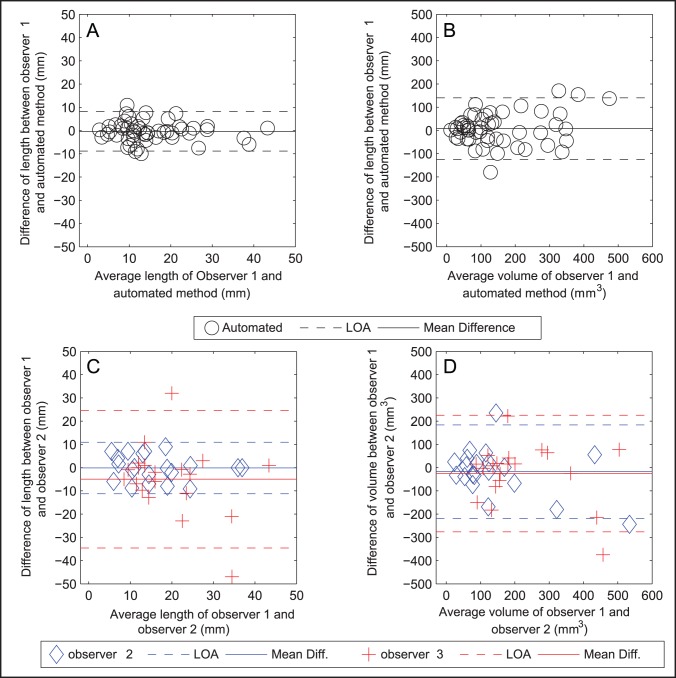
Bland-Altman plot comparing thrombus volume and length as measured by observer 1 and the automated method (A, B), and for all observers (D, C).

We included 78 consecutive patients with thin slice CTA and NCCT. In two patients the NCCT and/or CTA scans were incomplete. Fifty-seven (77%) of the remaining 74 patients had a visible hyperdense thrombus on NCCT. Four patients were randomly included in the training set, the remaining 53 constituted the test set. Forty-one percent of the patients were female and the mean age was 63.4 (±11.8) years. The method successfully segmented the thrombus for all patients. Figure 3 shows a scatter plot of thrombus length and volume measurements of observer1 versus observers 2, 3, and the automated method. Table 2 shows the results of the statistical analysis of the volume and length measurements. There was a high agreement between observer1 and the automated method with ICC of 0.89 and 0.84 for length and volume respectively. These values were similar to the agreement between observer 1 and 2 with ICC of 0.90 and 0.85 respectively and exceeded the agreement between observer 1 and 3. For brevity, we omitted the comparison of the automated method with observer 2 and 3, which were somewhat lower but comparable to the agreement with observer 1. Figure 4 shows the results of the Bland-Altman analysis, the agreement between observer 1 and the automated method was similar to the interobserver agreement. This figure also shows that there are couples of strong outliers in the comparison of observer 1 and 3. The automated segmentation required 5–7 minutes, while the manual delineations took in average 20 minutes per patient.

**Table 2:** Comparison of volume and length measurements for the automated method and observers.

	Average ± SD	(min, max)	N	Average paired difference ± SD	Limits of agreement, upper/lower limit	ICC
Length (mm)						
Observer 1	15.1±9.0	(2.0, 43.8)	53	−0.4±4.3	−8.8/7.7	0.89*
Automated	15.4±9.3	(2.7, 42.8)				
Observer 1	15.9±9.0	(3.0, 36.9)	20	−0.1±5.7	−11.2/10.9	0.90*
Observer 2	16.1±9.8	(2.0, 36.9)				
Observer 1	17.1±10.0	(6.0, 43.8)	20	−5.0±15.1	−34.7/24.6	0.31
Observer 3	22.1±13.9	(4.0, 57.9)				
Volume (mm^3^)						
Observer 1	152±125	(12, 542)	53	7.5±67	−126/141	0.84*
Automated	145±110	(11, 406)				
1Observer 1	137±122	(13, 460)	20	−17±103	−218/185	0.85*
Observer 2	154±163	(12, 656)				
Observer 1	205±127	(15, 542)	20	−25±127	−340/224	0.61†
Observer 3	230±157	(68, 644)				

*p-value <0.001

† p-value <0.005.

## Discussion

In this study, a method for automated thrombus segmentation on CTA using information from the contralateral arterial segment was proposed. The method is relatively fast and requires minimal user input. The validation showed a high accuracy in automated length and volume measurement, comparable with the best interobserver agreement.

Current methods to measure thrombus characteristics are either based on manual or semi-automated measurement of hyperdense thrombus on non-contrast CT or on quantification of absence of contrast-enhanced lumen in CT angiography.

Most of these methods are based on direct measurement using a straightforward ruler tool available on a diagnostic workstation or visual scoring. More advanced length measurements have been performed on hyperdense middle cerebral thrombus on NCCT [6]. The interobserver agreement found in our study is somewhat smaller than the values reported in a previous study with a mean deviation from the reference values and limits of agreement of 0.1 mm ±0.7 mm for length [7]. This may be explained by the smaller thrombi that were present in their patient population; all thrombi were smaller than <20.0 mm and <150 mm^3^. In contrast to their approach, our method does not rely on intensities of high-density thrombus and therefore is suitable for segmentation and measurement of hypodense thrombus. However, we could not validate the accuracy of automated measurement of low density thrombus owing to the difficulty of manual segmentation. Fifty seven (77%) patients had a visible thrombus on NCCT. This finding is rather high compared to the literature, which suggests that high-density thrombi are more often observed in thin slice reconstructions [20].

This study has some limitations. Because the trial was still ongoing, for many patients not all image data was available in the image database at the time of this study. Only for 74 out of 230 patients, thin slice (< = 2.5 mm) NCCT and CTA data were available. As a result, further studies on larger cohorts are necessary to confirm our results in other populations. Moreover, this study does not determine predictive value of the different parameters of the thrombus (length, volume or density) as the patient outcome was not available at the current stage of the trial. The accuracy of the method could be limited by the large variety of scanner manufacturers in this study. As a result, imaging parameters such as reconstruction parameters and slice thickness varied considerably. However, since our method successfully segmented the thrombi for all used scans, it appears to be robust to these variations. Additionally, in our population we did not observe large variations in the protocols.

Another limitation is the lack of a gold standard for the thrombus characteristics. Theoretically, measurements of the surgically removed thrombus could serve as gold standard. However endovascular techniques disrupt the integrity of the thrombus. Therefore, we used the current reference standard (manual delineation) for assessing the accuracy of the automated method. This comparison indicated that the difference between the method and the manual delineation is within the range of interobserver disagreement. Because of the large effort required for manual assessment, the number of measurements of observer 2 and 3 was rather limited.

Because manual segmentation of low-density thrombus on NCCT was difficult even for very experienced neuroradiologists, accuracy of automated measurement could not be validated for these thrombi. For segmentation of low density thrombi, good collateral retrograde filling may be required. Current literature suggests that good collateral retrograde filling of an occluded artery is common but not always the case [5], [12]. Poor retrograde filling may result in overestimation of the thrombus on CTA. However, due to the intensity based region growing segmentation used in our pipeline, our method is not sensitive for poor retrograde filling. Actually the large agreement between the automated method using CTA and the manual delineation on NCCT indicates that the method indeed is robust for delayed retrograde filling. In future studies, low-density thrombus segmentation could be validated by comparison with other measurements such as clot-burden score.

In our method, we assumed symmetry in order to retrieve the radius of the occluded segment for background masking. However, the occluded vascular tree and the contralateral vascular tree are not perfectly symmetric. To deal with this asymmetry, we used an elastic, non-rigid registration, which allows for differences in length, shape, and location of bifurcation. Because the accuracy of the method in 53 cases is in the range of the interobserver variability, we believe that the non-rigid registration accurately corrects for these contralateral differences. Yet, little is known about the degree of symmetry of radii of the proximal intracranial arteries. Violation of this assumption may result in errors. This could explain the larger limit of agreement for volume measurements compared to length measurement.

In three cases, we observed a strong disagreement between observer 1 and observer 3. Retrospective analysis of these three outliers revealed that this could be explained by high hematocrit level and vascular calcifications. Arteries in image datasets of patients with elevated hematocrit are known to mimic high density thrombi in CT images because of the higher attenuation on CT [20]. It is not uncommon to exclude patients with high hematocrit in thrombus analysis [4]. We believe that the observers disagreed on the interpretation of these hyperdense arteries. By excluding patients with high-hematocrit levels, as was done in previous studies, the interobserver agreement significantly improved to ICC of 0.85 and 0.78 for length and volume respectively. These values are in the range of the ICC of observer 1 and observer 2.

Image-supported treatment decision for patients with acute ischemic stroke was recently, recommended before treatment initialization by the American Society of Neuroradiology as well as number of current studies aim to it [4], [12], [21], [22]. Predictors of successful recanalization are likely to differ for mechanical thrombectomy and intra-arterial fibrinolysis. Thrombus characteristics have been suggested to be a determinant for inclusion for IV treatment. Indeed, the thrombus length might particularly be useful as a parameter to better identify patients who are unlikely to benefit from IVT [21]. In an ongoing randomized controlled trial, it is currently uses as an inclusion criterion [http://clinicaltrials.gov/show/NCT01429350]. Whereas thrombus burden was an important predictor for the success of IV treatment, mechanical thrombectomy may be more influenced by other target characteristics like thrombus branching and vessel curvature [23].

The presented method has the potential to support current clinical research and to be introduced in clinical practice to support treatment decisions for patients with acute ischemic stroke. With this method, multivariate analysis of many thrombus characteristics (length, volume, density, curvature, etc.) could be performed to relate these to stroke subtype, response to treatment, and patient outcome measures. This way, thrombus characteristics with the most prognostic value could be determined. However, further prospective studies will have to demonstrate the feasibility of this method in acute clinical setting and determine the predictive value of imaging features of thrombus on CTA.

## Conclusions

We developed and validated an automated tool performing thrombus length and volume measurements. We have shown that for high-density thrombi, this method is highly accurate, with accuracies comparable to interobserver agreements. Furthermore, this method was shown to be able to segment thrombi even for cases with high hematocrit level or calcifications, and thus could facilitate segmentation of hypodense thrombi. However, further studies on larger cohorts are necessary to confirm our results in larger and different populations.
